# Lithuanian Study on *COL4A3* and *COL4A4* Genetic Variants in Alport Syndrome: Clinical Characterization of 52 Individuals from 38 Families

**DOI:** 10.3390/ijms26157639

**Published:** 2025-08-07

**Authors:** Agne Cerkauskaite-Kerpauskiene, Milda Navickaite, Judy Savige, Gabija Mazur, Deimante Brazdziunaite, Karolis Azukaitis, Gerda Slazaite, Arvydas Laurinavicius, Marius Miglinas, Vija Vainutiene, Rasa Strupaite-Sileikiene, Ausrine Misevice, Vaiva Mickeviciene, Rimante Cerkauskiene

**Affiliations:** 1 Vilnius University, Faculty of Medicine, Institute of Clinical Medicine, LT-03101 Vilnius, Lithuania; karolis.azukaitis@santa.lt (K.A.); marius.miglinas@santa.lt (M.M.); vija.vainutiene@santa.lt (V.V.); rasa.strupaite@santa.lt (R.S.-S.); ausrine.misevice@santa.lt (A.M.); vaiva.mickeviciene@santa.lt (V.M.); rimante.cerkauskiene@santa.lt (R.C.); 2 Vilnius University, Faculty of Medicine, LT-03101 Vilnius, Lithuania; milda.navickaite00@gmail.com (M.N.); gerda.slazaite@mf.stud.vu.lt (G.S.); 3 The University of Melbourne, Department of Medicine (MH and NH), Royal Melbourne Hospital Level CSB, Parkville, VIC 3050, Australia; j.savige@unimelb.edu.au; 4 Centre for Medical Genetics, Vilnius University Hospital Santaros Klinikos, LT-08406 Vilnius, Lithuania; gabija.mazur@santa.lt; 5 Vilnius University, Faculty of Medicine, Institute of Biomedical Sciences, LT-03101 Vilnius, Lithuania; deimante.brazdziunaite@santa.lt (D.B.); arvydas.laurinavicius@vpc.lt (A.L.)

**Keywords:** Alport syndrome, *COL4A3*, *COL4A4*, genotype–phenotype correlation

## Abstract

Variants in *COL4A3* and *COL4A4* cause autosomal dominant and recessive Alport syndrome, yet data on their distribution and clinical expression in different populations remain limited. This study investigated genotype–phenotype correlations and the distribution of *COL4A3*/*COL4A4* variants in a Lithuanian Alport syndrome cohort. A total of 221 individuals from Lithuania were analyzed for *COL4A3* and *COL4A4* variants using either next-generation sequencing or Sanger sequencing in order to assess variant distribution and associated clinical features. Only individuals with pathogenic, likely pathogenic, or uncertain significance variants were included. Fifty-two individuals (38 index cases) with pathogenic, likely pathogenic, or variants of uncertain significance were identified, as follows: forty-eight were heterozygous, four had autosomal recessive, and four had digenic Alport syndrome. *COL4A3* variants were found in 9.5% (21/221) and *COL4A4* in 17.6% (39/221). Among the 28 identified variants, 18 were novel. Glycine substitutions (n = 8) were the most frequent and associated with worse kidney outcomes and increased hearing abnormalities. Hematuria was diagnosed significantly earlier than proteinuria (*p* = 0.05). Most individuals with autosomal dominant Alport syndrome had normal kidney function (eGFR > 90 mL/min/1.73 m^2^), while those with autosomal recessive Alport syndrome had more severe disease. Kidney failure occurred in 2/4 (50%) autosomal recessive Alport syndrome and 2/48 (4%) autosomal dominant Alport syndrome cases. A significant inverse correlation was found between eGFR and age in proteinuric individuals (r = –0.737; *p* = 0.013). This study expands knowledge of Alport syndrome in the Lithuanian population and contributes novel variant data to the global Alport syndrome genetic database.

## 1. Introduction

Alport syndrome (AS) is an inherited kidney disorder characterized by microhematuria, progressive kidney dysfunction, and often sensorineural hearing loss and ocular abnormalities [1]. It is the second-most common genetic cause of kidney failure (KF), responsible for up to 2% of all KF cases [2,3]. AS is caused by pathogenic variants in *COL4A3*, *COL4A4*, or *COL4A5* (*COL4A3–A5*) genes, which encode the α3, α4, and α5 chains of type IV collagen—a key component of the glomerular, cochlear, and ocular basement membranes [4,5,6]. Disruption in these chains affects collagen synthesis, assembly, and function, leading to organ-specific damage [7].

AS may follow X-linked (*COL4A5*), autosomal recessive, or autosomal dominant (*COL4A3/4*) inheritance patterns [5,7,8]. Digenic inheritance involving two of the *COL4A3–A5* genes has also been described [9]. While AS prevalence is estimated at 1 in 5000 to 1 in 53,000 individuals [10,11], newer studies suggest that X-linked AS (XLAS) may occur in at least 1 in 2000 and autosomal dominant AS (ADAS) in up to 1 in 100 individuals [12]. These findings highlight the likelihood of underdiagnosed or asymptomatic carriers of pathogenic variants [13].

Clinical severity varies depending on the gene, sex, and type of variant [7,14,15,16,17,18,19]. Comprehensive clinical assessment, including family history, persistent hematuria, extrarenal signs, and molecular analysis of *COL4A3–A5*, is essential for diagnosis [13]. In this study, we focused on core clinical features typically associated with *COL4A3-* and *COL4A4-*related AS to assess genotype–phenotype correlations. These included hematuria, proteinuria, estimated glomerular filtration rate (eGFR), hearing loss, and ocular abnormalities. These phenotypic characteristics were selected based on established diagnostic criteria and their relevance to disease progression and severity, as documented in previous literature.

Global data on AS remain limited, particularly regarding *COL4A3* and *COL4A4*. This study aims to describe the genotype–phenotype relationship and the distribution of *COL4A3* and *COL4A4* variants in a Lithuanian cohort—marking the first such analysis in this population.

## 2. Results

### 2.1. General Genetic Findings

NGS and Sanger sequencing of the *COL4A3*, *COL4A4*, and *COL4A5* genes in 221 individuals from 120 families identified 28 distinct *COL4A3–A4* variants in 52 individuals from 38 families. Although *COL4A5* variants were also detected, the analysis focused on *COL4A3* and *COL4A4*. *COL4A3* variants were identified in 32% (12/38) of unrelated families while *COL4A4* variants in 50% (19/38). Among the 28 variants, 12 (9 novel and 3 known) were in *COL4A3* and 16 (9 novel and 7 known) in *COL4A4*. A summary of the *COL4A3* and *COL4A4* genetic changes found in the Lithuanian cohort is shown in Table 1, Table 2 and Table S1. Novel variants have been described in detail [20].

In this study, three different types of genetic variants including missense 85% (24/28), frameshift 3.5% (1/28), and splice site 10.7% (3/28) variants in *COL4A3–A4* genes were identified. Different missense variants including Gly substitutions (*n* = 16) and non-Gly substitutions (other amino acid change, *n* = 8) were the most frequent types of genetic changes in all *COLA4A3-A4* genes and accounted for 90.3% of all individuals with AS. The amino acid changes in different Gly substitutions distributed as follows 50% arginine (Arg, R), 25% valine (Val, V), 18.75% glutamic acid (Glu, E), 12.5% serine (Ser, S). Some individuals carried multiple variants within or across these genes and were included due to their potential clinical significance. 

### 2.2. Autosomal Dominant Alport Syndrome

In total, 48 (82.8%) individuals with one heterozygous *COL4A3* or *COL4A4* variant, thus ADAS, were identified and included 24 males. In ADAS group, the probands represented 54.2% (*n* = 26) of individuals, with the remaining 45.8% (*n* = 22) identified during family screening. Two males were incidentally diagnosed with AS through genetic testing during screening for autosomal dominant polycystic kidney disease, with no pathogenic variants found in the *PKD1* or *PKD2* genes. Among ADAS patients, 33.3% (*n* = 16) were found to be heterozygous for *COL4A3* genetic variants, while 66.7% (*n* = 32) were heterozygous for *COL4A4* genetic variants (Table 3 and Table S2).

The average age at diagnosis was 24 ± 18.7 for males and 37.7 ± 20.3 for females. In this research, no notable distinctions in kidney function, hearing abnormalities, ocular lesions, or other characteristics were identified across genders (*p* = 0.13) or different *COL4A3* or *COL4A4* genes (*p* = 0.56). Therefore, genotype–phenotype correlations of females and males with ADAS and different *COL4A3* and *COL4A4* genes were considered together in further analysis. KF was linked to one non-Gly substitution (c.4421T>C, p.Leu1474Pro) in the *COL4A3* gene and one splice site variant (c.594+1G>A) in *COL4A4* (Table 4 and Table S3).

**Figure 1:**
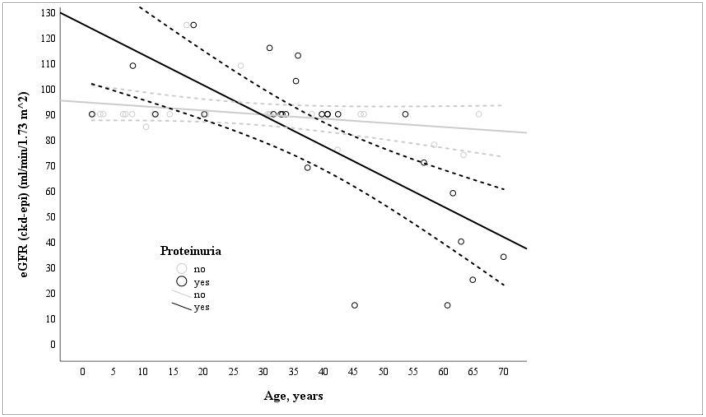
Correlation of age and eGFR levels in proteinuric (*n* = 25; r = −0.737; *p* = 0.013) and non-proteinuric (*n* = 23; r = −0.321, *p* = 0.135) groups of individuals with ADAS at single time points.

All 48 ADAS heterozygotes experienced hematuria, whereas proteinuria was detected in 26 individuals. However, only one patient developed nephrotic-range proteinuria and chronic kidney disease (CKD) stage 2. Among the individuals with ADAS and proteinuria, more than half maintained normal kidney function. In contrast, only two individuals developed KF at the ages of 55 and 44. A significant association was identified between lower eGFR levels and older age in individuals with proteinuria (*n* = 25; r = −0.737; *p* = 0.013), while no significant correlation was observed in individuals without proteinuria (*n* = 23; r = −0.321, *p* = 0.135) (Figure 1).

Similar eGFR rate levels were observed among individuals with different types of genetic variants in *COL4A3–A4* genes, and there were no significant differences detected between the groups.

Kidney biopsy was performed in 21 individuals with ADAS, with the mean age being 43.0 ± 15.1. Thin basement membrane (*n* = 19) and foot process effacement (*n* = 19) were the most common characteristics described in electron microscopy (EM). Lamellation or thickening of the glomerular basement membrane (GBM) was observed in 28.6% (6/21) of individuals at a mean age of 49 ± 17.4 years and was associated with higher eGFR values compared to those with focal segmental glomerulosclerosis (FSGS) (eGFR 85.5 ± 38.8 mL/min/1.73 m^2^, *n* = 6 vs. 58.6 ± 31.4 mL/min/1.73 m^2^, *n* = 8). The general findings from kidney biopsy in individuals with ADAS are shown in Table S4. 

Eight (16.7%) individuals with ADAS exhibited bilateral sensorineural hearing abnormalities, which were detected at a mean age of 46.5 ± 11.8 years. Eight (16.7%) individuals with ADAS exhibited ocular lesions specific to AS, which were detected at a mean age of 16.6 ± 17.3 years. The diagnosis of ocular abnormalities was higher in individuals with ADAS who were older than 6.6 years (*p* = 0.02, 95% CI [1.6–216.1]).

### 2.3. Autosomal Recessive Alport Syndrome

In total, four (3.5%) individuals from three different families with ARAS were identified in the study, including one male. ARAS was genetically confirmed in three probands, which accounted for 75% of the cases, and one of their relatives, constituting 25% of the cases. Three individuals were diagnosed based on clinical features alone or with a kidney biopsy, while one female was referred for genetic analysis due to her twin sister’s positive AS. The general characteristics and genotype–phenotype correlations of individuals with ARAS can be found in Tables S5 and S6. Three individuals were compound heterozygotes for genetic variants in *COL4A3* (*n* = 2) or *COL4A4* (*n* = 1), while one individual was homozygous for genetic variants in *COL4A4*. One variant was categorized as pathogenic, three as likely pathogenic, and four as VUS. Hematuria and proteinuria were observed in all four individuals, with two of them developing KF at the ages of 32 and 33, with homozygous variant p.Ala607Val in *COL4A4* and compound heterozygous variants p.Pro1568Ser and p.Gly1083Arg in *COL4A3*, respectively. Three females with ARAS underwent kidney biopsy at a mean age of 30.0 ± 3.2. All three females exhibited proteinuria exceeding 1 g/L at the time of the KB, and two of them had abnormal kidney function (median eGFR was 50.0, with a minimum of 45 and a maximum of 55 mL/min/1.73 m^2^). The general findings of the KB are listed in Table S7. All four individuals exhibited sensorineural deafness, with the youngest being 7 years old. Hearing loss was detected in both patients with KF as well. Two individuals with pathogenic variants in the *COL4A3* gene had ocular abnormalities at the ages of 7 and 32. One of these patients was diagnosed with KF at the age of 32, while the other maintained normal kidney function.

### 2.4. Digenic Alport Syndrome

Four (3.5%) unrelated individuals with suspected digenic AS were identified in the study, including three females. The general characteristics and genotype–phenotype correlations of individuals with digenic AS can be found in Tables S8 and S9. All three females had a gene combination of *COL4A4* and *COL4A5*, while *COL4A3* and *COL4A4* genetic variants were identified in the male. All variants in *COL4A3* and *COL4A4* were classified as VUS, while changes in *COL4A5* were classified as pathogenic (*n* = 1) or likely pathogenic (*n* = 2). Three missense alterations in *COL4A4* were detected, and one of them had glycine substitutions. One splice site variant in *COL4A4* and one missense variant in *COL4A3* were found as well. Hematuria and proteinuria were detected in all four individuals, with three of them exhibiting normal kidney function (eGFR greater than 90 mL/min/1.73 m^2^). Only one female developed CKD stage 2 with her proteinuria reaching over 50 mg/mmol. Two females and one male underwent kidney biopsy at the mean ages of 25.5 ± 16.3 and 40, respectively. The general findings of kidney biopsy in individuals with digenic AS are shown in Table S10. Hearing loss and ocular abnormalities were detected only in one female, who also developed the 2nd stage of CKD.

## 3. Discussion

Identifying pathogenic variants in *COL4A3* and *COL4A4* is critical for diagnosing and managing AS. Although over 5000 variants have been reported [21], many remain undiscovered [22]. In our cohort of 221 individuals from 120 families, we identified 28 different *COL4A3–A4* variants. Sixty-four percent of variants in *COL4A3–A4* genes were novel [20], while others (36%) were previously described and found globally, originating from Central/East Europe around 750–900 years ago [23]. These findings broaden knowledge on AS in Lithuania and raise the estimated prevalence to at least 1 in 106 individuals, though actual rates are likely higher due to undetected variants [12].

It is important to emphasize that our analysis represented a cohort-specific interpretation and did not involve formal variant reclassification. In several cases, individuals carrying variants classified as VUS exhibited clinical features consistent with AS. This may suggest a potentially pathogenic role, possibly influenced by epigenetic factors or other genetic modifiers.

ADAS cases were mainly identified through family screening (71%), while ARAS diagnoses followed abnormal kidney biopsies (40.7%). This reflects ARAS’s more severe, early-onset phenotype, often requiring invasive diagnostics, while ADAS typically presents milder, later-onset symptoms. These results highlight the importance of family-based screening for early detection and renoprotective therapy initiation [24,25,26,27].

Clinical manifestations of AS are genotype-dependent [28]. Hematuria consistently appeared before proteinuria in all AS types. KF occurred in 50% of ARAS and 4% of ADAS cases, aligning with ARAS’s known severity. Proteinuria and male sex were risk factors for CKD progression, in line with findings in broader CKD populations [29,30,31,32]. Our data support using proteinuria as a key prognostic marker and rationale for early intervention [13,33]. In our cohort, the inverse correlation between estimated glomerular filtration rate and age among individuals with proteinuria suggests progressive kidney function decline, highlighting the prognostic value of proteinuria and the importance of initiating early therapeutic interventions to delay disease progression.

ADAS displays wide phenotypic variability. Only 2 of 48 individuals developed KF, both with additional risk factors (e.g., delayed diagnosis, FSGS, and severe proteinuria). One notable case involved a hypomorphic *COL4A3* variant (p.Leu1474Pro) in a male who progressed to KF earlier than a female with a pathogenic *COL4A4* splice-site variant, despite the literature suggesting non-missense variants typically result in more severe outcomes [34]. This case suggests that compound or undetected variants may modulate disease severity.

Extrarenal manifestations were observed in 17% of ADAS cases (ocular or auditory), consistent with previous studies [33,35]. Although less frequent than in ARAS, these findings support the heterogeneity of ADAS and the potential presence of additional undiagnosed variants or digenic inheritance in more severe cases.

Data on ARAS genotype–phenotype correlations remain limited [36,37,38]. In our study, two women with ARAS developed KF in their early 30s. One pair of dizygotic twins with identical compound heterozygous *COL4A3* variants showed differing severity, suggesting environmental or epigenetic modifiers. One ARAS case with a homozygous *COL4A4* variant arose from a consanguineous family, underscoring the impact of genetic background on disease risk. Another boy with compound heterozygous *COL4A4* variants showed early-onset hematuria and proteinuria but preserved kidney function at age 12, further illustrating variability. All ARAS individuals had hearing loss, and half had ocular abnormalities, consistent with severe phenotypes similar to males with XLAS [21].

Our findings reinforce that ARAS and digenic AS are frequently underdiagnosed due to lack of family history and overlapping phenotypes. Both genders with ARAS show comparable risk of KF and extrarenal symptoms.

Limitations of this study include its single-center design, a small sample size for kidney failure (KF) cases, limited eGFR data, and potential bias due to missing data, inclusion of young children, and related individuals. Proteinuria and eGFR were measured at a single time point without longitudinal follow-up, limiting our ability to assess disease progression over time. Interpretations of genetic variants are specific to our study population and should not be generalized. Additionally, the absence of functional validation and the potential influence of epigenetic or other unidentified factors limit the broader applicability of our conclusions.

## 4. Materials and Methods

### 4.1. Study Design and Participants

A cross-sectional study between 2016 and 2022 was performed. Two hundred and twenty-one individuals (aged 2 months to 82 years) with suspected AS [13] or at-risk relatives were assessed by nephrologists at Vilnius University Hospital Santaros Klinikos (VUH SK), Vilnius, Lithuania, and provided blood samples for DNA analysis. Clinical, demographic, and family history data were collected at the initial visit. The study was approved by the Vilnius Regional Biomedical Research Ethics Committee (BioAlport, No. 158200-16-857-367).

### 4.2. Genetic Analysis 

Genetic testing was conducted using next-generation sequencing (NGS) or Sanger sequencing. The genetic methodology is described in detail in our previous publication [20] and in the article available at https://www.mdpi.com/1648-9144/61/4/597 (accessed on 27 July 2025). Most samples (85%) were analyzed at Centogene (Rostock, Germany) and the rest at the Centre for Medical Genetics, VUH SK (Vilnius, Lithuania). We examined variants with a minor allele frequency <1% and known pathogenic mutations in *COL4A3*, *COL4A4*, and *COL4A5*. Individuals with pathogenic (P), likely pathogenic (LP), or VUS in *COL4A3* or *COL4A4* were included. While VUS do not confirm AS, they were retained due to clinical relevance and reassessed based on available clinical and genetic data.

### 4.3. Study-Based Interpretation of Pathogenicity of Genetic Variants

Variant classification followed ACMG guidelines, using databases (ClinVar, InterVar, Franklin, Centogene) and in silico tools (PPT, SIFT, MutationTaster, Align-GVGD). Genetic variant interpretation in this study was based solely on clinical evaluation, disease course, biopsy results, sex, age, and familial history observed in our patient cohort. We excluded other kidney pathologies and co-morbidities to support a more accurate phenotype–genotype correlation. VUS were upgraded to pathogenic or likely pathogenic if accompanied by features such as nephrotic-range proteinuria, reduced eGFR (<60 mL/min/1.73 m^2^), CKD stages III–V, extrarenal signs (hearing/ocular), or affected relatives with CKD/KF. The absence of Alport-specific features in multiple carriers and the presence of only isolated hematuria with mild albuminuria in a single individual without family history of KF or progression supported interpretation of the variant from VUS or pathogenic to likely benign or to remain a VUS.

### 4.4. Clinical Evaluation 

Participants with a genetic diagnosis of AS underwent follow-up evaluation by nephrologists, including assessment of hematuria (spot urine), proteinuria (spot urinary protein-to-creatinine ratio [uPr/uCr, PCR] and albumin-to-creatinine ratio [uA/Cr, ACR] in mg/mmol, per KDIGO 2020/2021 guidelines), and CKD staging using eGFR (CKD-EPI for adults, Schwartz 2009 for children). GFR and proteinuria values were evaluated at a single time point. Arterial hypertension was defined as blood pressure > 130/90 mmHg in adults (2017 ACC/AHA guideline) or ≥95th percentile for age, sex, and height in children (2016 European Society of Hypertension guidelines in children and adolescents). Individuals were referred for ophthalmologic evaluation [19]. AS-related ophthalmopathy was considered present if one or more of the specified criteria were met. Audiometric testing was performed in a soundproof booth (Interacoustics AC40), using air and bone conduction thresholds across frequencies of 125–8000 Hz. As early ocular and hearing abnormalities are often under-recognized, we recorded the age at which these abnormalities were first diagnosed.

### 4.5. Statistical Analysis 

Data were analyzed using SPSS 27.0. Continuous variables were presented as the mean ± standard deviation or median with interquartile range, depending on the distribution. Categorical data are expressed as frequencies and percentages. Appropriate statistical tests, including ANOVA, Kruskal–Wallis, chi-square, Fisher’s exact test, and Mann–Whitney, were employed to compare variables. A significance level of *p* ≤ 0.05 was considered statistically significant. Due to the limited number of individuals with kidney failure, kidney survival analysis using Kaplan–Meier tests was not feasible. It is important to acknowledge that a direct comparison between ARAS and ADAS was not feasible due to the smaller cohort sizes of ARAS compared to ADAS.

### 4.6. Data Availability

The original findings presented in the study have been made publicly accessible and are located in the ClinVar Database, specifically under the accession SUB11112191 (SCV002098105–SCV002098129). However, it should be noted that the authors acknowledge a partial unavailability of raw data within this study. This is primarily due to the Centogene laboratory’s imposed time restriction, wherein variants and their corresponding raw data older than 5 years are not stored. The limited accessibility of data represents a potential significant constraint within the study.

## 5. Conclusions

In summary, 12 different *COL4A3* and 16 different *COL4A4* genetic variants were identified by NGS or Sanger method testing in 221 individuals from 120 families with suspected AS in a single-center Lithuanian cohort. The majority of the affected individuals had ADAS (82.8%) but ARAS (3.5%) and digenic disease (3.5%) were also identified. Despite the genetic variant, individuals with ADAS and digenic AS mostly retained normal kidney function, while individuals with ARAS developed more severe kidney disease. Ocular abnormalities and hearing loss were more severe and more frequent in individuals with ARAS compared to ADAS as well. However, Alport syndrome caused by *COL4A3* and *COL4A4* changes should always be regarded as potentially serious. Overall, it is the first such research of AS in Lithuania, and the results of this study expand the number of identified *COL4A3* and *COL4A4* variants. It increases our understanding of genotype–phenotype correlations in AS, which play a crucial role in diagnosing and prognosticating for individuals with AS.

## Figures and Tables

**Table 1:** Classification of 12 different *COL4A3* genetic variants.

GV in COL4A3	Type (GV Identification Number)	M/F with GV	No. of Diff. Fam.	ACMG Criteria	Our Study Interpretation
c.520G>A (p.Gly174Arg) ^b^	Gly subs. ^▲^ (19)	1M/1F	1	LP (PM1, PM2, PM5, PP3)	LP
c.898G>A (p.Gly300Arg) ^a^	Gly subs. ^▲^ (22)	2M/2F	2	LP (PM1, PM2, PP3, PP5)	VUS
c.4702C>T (p.Pro1568Ser) ^b^	Non-Gly subs. (23)	2F *	1	VUS (PM2, PP3)	P *
c.3247G>C (p.Gly1083Arg) ^b^	Gly subs. ^▲^ (24)	2F *	1	LP (PM1, PM2, PM5, PP3)	P *
c.3499G>A (p.Gly1167Arg) ^a^	Gly subs. ^▲^ (29)	2F	1	P (PM1, PM1, PM2, PM5, PP3, PP5)	LP
c.2711G>T (p.Gly904Val) ^b^	Gly subs. ^▲^ (31)	1M	1	LP (PM1, PM2, PM5, PP3)	LB
c.4717G>A (p.Gly1573Ser) ^b^	Gly subs. (35)	1M/1F	1	LP VUS (PM2, PP3)	LB
c.416G>A (p.Gly139Glu) ^b^	Gly subs. ^▲^ (37)	1F	1	LP (PM1, PM2, PM5, PP3)	LP
c.1021C>T (p.Arg341Cys) ^b^	Non-Gly subs. ^▲^ (38)	1M *	1	VUS (PM1, PM2)	LP *
c.593G>T (p.Gly198Val) ^b^	Gly subs. ^▲^ (39)	1M	1	LP (PM1, PM2, PP3)	LP
c.2188G>C (p.Gly730Arg) ^b^	Gly subs. ^▲^ (43)	1F	1	LP (PM1, PM2, PP3)	LB
c.4421T>C (p.Leu1474Pro) ^a^	Non-Gly subs. (44)	2M	1	LB (BS1, BS2, BP6, PP5, PP3)	Hypomorphic

* Including individuals with ARAS and digenic inheritance. No.—number; M—male; F—female; P—pathogenic; LP—likely pathogenic; VUS—variant of uncertain significance; LB—likely benign; subs.—substitution; Gly—glycine; GV—genetic variant; diff—different; fam—families. ^a^ Known genetic variant. ^b^ Novel genetic variant. ^▲^ Substitution in collagenous domain.

**Table 2:** Classification of 16 different *COL4A4* genetic variants.

GV in *COL4A4*	Type (GV Identification Number)	M/F with GV	No. of Diff. Fam.	ACMG Criteria	Our Study Interpretation
c.657+2dup ^b^	Splice site (5)	1F	1	LP (PVS1, PM2, PP5)	LB
c.4151C>T (p.Ala1384Val) ^a^	Non-Gly subs. ^▲^ (7)	2F *	2	VUS (PM1, PM2, BP4)	LP *
c.594+1G>A ^b^	Splice site (9)	3M */3F	2	LP (PVS1, PM2, PP5)	LP *
c.1579G>T (p.Gly527Cys) ^a^	Gly subs. ^▲^ (10)	2M	1	P (PM1, PM2, PM5, PP3)	LP
c.1987G>C (p.Gly663Arg) ^b^	Gly subs. ^▲^ (16)	2M	1	LP (PM1, PM2, PP3)	LB
c.-101-4A>G ^a^	Splice site (20)	1F	1	VUS (PM2, PP3)	LB
c.1389del (p.Asn464Thrfs*7) ^a^	Frame shift (25)	3M/1F	2	P (PVS1, PM1, PM2, PP5)	LP
c.4910G>A (p.Arg1637Gln) ^b^	Non-Gly subs. (27)	1F *	1	VUS (PM2, BP4)	LP *
c.1820C>T (p.Ala607Val) ^b^	Non-Gly subs. ^▲^ (28)	2F	2	VUS (PM1, PM2)	LP
c.2756A>G (p.Glu919Gly) ^b^	Non-Gly subs. ^▲^ (32)	1M/1F	1	VUS (PM1, PM2, BP4)	LB
c.4315G>A (p.Gly1439Ser) ^b^	Gly subs. ^▲^ (36)	2M/1F	1	LP (PM1, PM2, PM5)	LB
c.3044G>A (p.Gly1015Glu) ^b^	Gly subs. ^▲^ (40)	3M	1	LP (PM1, PM2, PP3)	LB
c.3451G>A ^a^ (p.Gly1151Arg)	Gly subs. ^▲^ (42)	1F	1	LP (PM1, PM2, PP3)	LP
c.5045G>A ^a^ (p.Arg1682Gln)	Non-Gly subs. (15)	2F	2	VUS (PM2, PP3)	LP
c.2347G>A ^b^ (p.Gly783Arg)	Gly subs. ^▲^ (41)	1F/2M	1	LP (PM1, PM2, PP3)	LP
c.2996G>A ^a^ (p.(Gly999Glu)	Gly subs. ^▲^ (11)	1F	1	B (PM1, BP4, BP6, BS1, BS2)	LB

* Including individuals with ARAS and digenic inheritance. No.—number; M—male; F—female; P—pathogenic; LP—likely pathogenic; VUS—variant of uncertain significance; LB—likely benign; subs.—substitution; Gly—glycine; GV—genetic variant; diff—different; fam—families. ^a^ Known genetic variant. ^b^ Novel genetic variant. ^▲^ Substitution in collagenous domain.

**Table 3:** General characteristics of 48 individuals with ADAS depending on affected gene.

Demographic and Clinical Features	Total No. of Ind.	*COL4A3*het	*COL4A4*het	*p*-Value
Number of subjects, %,	48 (100.0)	16 (33.3)	32 (66.7)	0.06
Females, %,	24 (50.0)	8 (50.0)	16 (50.0)	1.00
Age at genetic diagnosis	33.2 ± 19.8	33.3 ± 18.9	33.2 ± 21.0	0.98
Probands, %	26 (54.2)	10 (62.5)	16 (50.0)	0.41
Dgn. of AS by CF & KB, %	18 (37.5)	7 (43.8)	11 (34.4)	0.53
Dgn. of AS by CF only, %	6 (12.5)	2 (12.5)	4 (12.5)	1.00
Dgn. of AS by FS, %	22 (45.8)	6 (37.5)	16 (50.0)	0.41
Hematuria, %	48 (100.0)	16 (100.0)	32 (100.0)	0.06
Age at dgn. of hematuria	27.0 ± 18.3	23.4 ± 16.3	28.8 ± 19.2	0.34
Proteinuria, %	25 (52.1)	10 (62.5)	15 (46.9)	0.31
Age at dgn. of Pro, %	23.0 ± 17.3	25.1 ± 14.2	37.9 ± 17.8	0.70
Nephrotic range Pro, %	1 (2.1)	0 (0.0)	1 (3.1)	0.24
CKD I, %	35 (72.9)	11 (68.8)	24 (75.0)	0.44
CKD II, %	7 (14.6)	4 (25.0)	3 (9.4)	
CKD III *, %	3 (6.3)	0 (0.0)	3 (9.4)	
CKD IV, %	1 (2.1)	0 (0.0)	1 (3.1)	
CKD V (KF), %	2 (4.2)	1 (6.3)	1 (3.1)	
Mean age at KF	49.5 ± 7.8	44.0 ± 0	55.0 ± 0	1.00
Arterial hypertension, %	18 (37.5)	6 (37.5)	12 (37.5)	1.00
RAAS inhibition, %	19 (40.4)	8 (50.0)	11 (35.5)	0.34
Kidney biopsy, %	21 (43.8)	9 (56.3)	12 (37.5)	0.22
Age at kidney biopsy	43.0 ± 15.1	38.0 ± 11.6	46.7 ± 16.9	0.12
Hearing abnormalities, %	8 (16.7)	4 (9.1)	4 (13.8)	0.41
Ocular lesions, %	8 (16.7)	1 (7.7)	7 (25.9)	0.24

No.—number; Pro—proteinuria; KF—kidney failure; KB—kidney biopsy; FS—family screening; CFs’—clinical features; CKD—chronic kidney disease; dgn.—diagnosis; Ind.—individuals. eGFR staging based on CKD-EPI: CKD I ≥ 90, CKD II 60–89, CKD IIIa 45–59, CKD IIIb 30–44, CKD IV 15–29, CKD V < 15 mL/min/1.73 m^2^ (KDIGO 2012). * CKD stage III was not divided into IIIa or IIIb and was counted together in our study, as it did not have a significant impact on our results or aims to the study.

**Table 4:** Genotype–phenotype correlations in individuals with ADAS.

Features	Total (%)	Missense Gly (%)	Non-Gly (%)	Frameshift (%)	Splice Site (%)
Hematuria	48 (100)	27 (100.0)	10 (100.0)	4 (100.0)	7 (100.0)
Pro	25 (52.1)	11 (40.0)	7 (70.0)	2 (50.0)	5 (71.4)
Nephrotic range Pro	1 (2.1)	0 (0.0)	1 (10.0)	0 (0.0)	0 (0.0)
CKD I	35 (72.9)	20 (74.1)	6 (60.0)	4 (100.0)	5 (71.4)
CKD II	7 (14.6)	5 (18.5)	2 (20.0)	0 (0.0)	0 (0.0)
CKD III *	3 (6.3)	2 (7.4)	1 (10.0)	0 (0.0)	0 (0.0)
CKD IV	1 (2.1)	0 (0.0)	0 (0.0)	0 (0.0)	1 (14.3)
CKD V	2 (4.2)	0 (0.0)	1 (10.0)	0 (0.0)	1 (14.3)
KB	21 (43.8)	7 (25.9)	8 (80.0)	2 (50.0)	4 (57.1)
TBM	18 (37.5)	7 (25.9)	7 (70.0)	2 (50.0)	3 (42.9)
TcGBM	6 (12.5)	2 (7.4)	3 (30.0)	0 (0.0)	1 (14.3)
FSGS	8 (16.7)	3 (11.1)	3 (30.0)	0 (0.0)	2 (28.6)
Hearing a.	8 (16.7)	4 (14.8)	2 (20.0)	0 (0.0)	2 (28.6)
Ocular a.	8 (16.7)	5 (18.5)	0 (0.0)	0 (0.0)	3 (42.9)
Total	48 (100.0)	27 (56.3)	10 (20.8)	4 (8.3)	7 (14.6)

Pro—proteinuria; CKD—chronic kidney disease; KB—kidney biopsy; TBM—thin basement membrane; TcGBM—thickened and lamellated GBM; FSGS—focal segmental glomerulosclerosis; a.—abnormalities. eGFR staging based on CKD-EPI: CKD I ≥ 90, CKD II 60–89, CKD IIIa 45–59, CKD IIIb 30–44, CKD IV 15–29, CKD V < 15 mL/min/1.73 m^2^ (KDIGO 2012). * CKD stage III was not divided into IIIa or IIIb and was counted together in our study, as it did not have a significant impact on the results or aims of our study.

## Data Availability

The online version of this article contains Supplementary Materials. Any additional information is available from the authors upon request.

## Supplementary Materials

The following supporting information can be downloaded at: https://www.mdpi.com/article/10.3390/ijms26157639/s1.

## Abbreviations

The following abbreviations are used in this manuscript:

ACMG	The American College of Medical Genetics and Genomics
ADAS	autosomal dominant Alport syndrome
AMP	Association for Molecular Pathology
ARAS	autosomal recessive Alport syndrome
AS	Alport syndrome
CKD	chronic kidney disease
CKD-EPI	Chronic Kidney Disease Epidemiology Collaboration
eGFR	estimated glomerular filtration rate
FSGS	focal segmental glomerulosclerosis
KB	kidney biopsy
KF	kidney failure
NGS	next-generation sequencing
VUS	variant of uncertain significance
XLAS	X-linked Alport syndrome
