# Supplementary material for: Lithuanian Study on COL4A3 and COL4A4 Genetic Variants in Alport Syndrome: Clinical Characterization of 52 Individuals from 38 Families
Source: Int J Mol Sci. 2025 Aug 7;26(15):7639. doi: 10.3390/ijms26157639 (PMC12347723; doi:10.3390/ijms26157639)
Supplement: Supplementary file 1 [file ijms-26-07639-s001.zip › ijms-3755155-supplementary.pdf]

SUPPLEMENTS

Table S1. Genetic characteristics and classification of *COL4A3* (NM_000091.5) and *COL4A4* (NM_000092.5) variants

Gene	Nucleotide change	Protein change	No of var	rsID	Coding impact	Described / Novel	ACMG criteria	ClinVar classification	CentoGene classification	Our study interpretation	Allele frequency (gnomAD v4.1.0)	REVEL	CADD	PP2	SIFT	MT	Align-GVGD
COL4A4	c.1579G>T	p.(Gly527Cys)	10	rs779930511	Gly subs. ▲	PubMed: 19129241	PM1, PM2, PM5, PP3	P(2);LP(1)	P	LP	N/a	0.987	28	pd	dlt	dc	C0
	c.-101-4A>G	-	20	rs147186690	splice site	PubMed: 25514610	PM2, PP3	VUS(2); LB(2);B(1)	VUS	LB	0.006427 (5973/ 929392)	N/a	21	N/a	N/a	N/a	N/a
	c.1389del	p.(Asn464Thrfs*7)	25	rs1576428862	frameshift	PubMed: 21196518	PVS1, PM1, PM2, PP5	P(1)/LP(1)	P	LP	0.000001239 (2/1613610)	N/a	N/a	N/a	N/a	N/a	N/a
	c.1820C>T[b]	p.(Ala607Val)	28	rs373916569	missense▲	Novel	PM1, PM2	VUS(2)	LP	LP	0.00001177 (19/1613630)	0.471	24	Pd	dlt	plm	C55
	c.3451G>A	p.(Gly1151Arg)	42	rs899227425	Gly subs. ▲	PMID: 35325889	PM1, PM2, PP3	LP(1); VUS(2)	LP	LP	0.000004957 (8/1613742)	0.945	26	pd	dlt	dc	C15
	c.657+2dup	-	5	rs2150877933	splice site	Novel	PVS1, PM2, PP5	LP(1)	LP	LB	0.000000619 (1/1613116)	N/a	N/a	N/a	N/a	N/a	N/a
	c.4151C>T	p.(Ala1384Val)	7	rs199911379	missense▲	PMID: 35419377	PM1, PM2, BP4	VUS(3); LB(1)	VUS	LP	0.00005205 (84/1613828)	0.132	13	bng	tlt	plm	C0
	c.594+1G>A	-	9	rs1553690565	splice site	Novel	PVS1, PM2, PP5	P(2);LP(3)	LP	LP	N/a	N/a	32	N/a	N/a	N/a	N/a
	c.5045G>A	p.(Arg1682Gln)	15	rs368404711	missense	PubMed: 17216251	PM2, PP3	P(2);LP(1); VUS(6)	N/a	LP	0.00007249 (117/ 1614058)	0.89	25	N/a	dc	dc	N/a
	c.1987G>C	p.(Gly663Arg)	16	N/a	Gly subs. ▲	Novel	PM1, PM2, PP3	N/a	N/a	LB	0.00000062 (1/1611972)	0.79	35	N/a	N/a	N/a	N/a
	c.2756A>G	p.(Glu919Gly)	32	rs753208968	missense▲	PubMed: 29854973	PM1, PM2, BP4	VUS(4)	VUS	LB	0.00003346 (54/1614020)	0.359	18	bn	dlt	plm	C0
	c.4315G>A	p.(Gly1439Ser)	36	N/a	Gly subs. ▲	Novel	PM1, PM2, PM5	P(1);LP(2); VUS(1)	LP	LB	0.000001859 (3/1613826)	0.722	26	pd	dlt	dc	C0

	c.3044G>A	p.(Gly1015Glu)	40	rs764323652	Gly subs.▲	PubMed: 28632965	PM1, PM2, PP3	P(1);LP(3); VUS(2)	N/a	LB	0.00006010 (97/1614008)	0.947	23	pat	pat	pat	N/a
	c.2347G>A	p.(Gly783Arg)	41	rs879255339	Gly subs.▲	Novel	PM1, BP4, BP6, BS1, BS2	VUS(1)	LP	LP	0.000009293 (15/1614038)	0.95	26	pd	dlt	dc	C15
	c.4910G>A	p.(Arg1637Gln)	27	rs937092831	missense	Novel	PM2, BP4	VUS(2)	VUS	LP	0.000009913 (16/1614026)	0.332	23	pd	tlt	dc	C0
	c.2996G>A	p.(Gly999Glu)	11	rs13027659	Gly subs.▲	PMID33:85421; PMID36:553470	PM1, PM2, PP3, PM5	VUS	VUS	LB	0.01663 (26837/ 1614116)	0.77	22	pd	pd	pd	pd
COL4A3	c.898G>A	p.(Gly300Arg)	22	rs772708743	Gly subs.▲	PubMed: 2557550	PM1, PM2, PP3, PP5	P(1);LP(4)	LP	VUS	0.00002045 (33/1613906)	0.998	29	pd	dlt	dc	C65
	c.3499G>A	p.(Gly1167Arg)	29	rs267606745	Gly subs.▲	PubMed: 11134255	PM1, PM2, PM5, PP3, PP5	P(3)	P	LP	0.000008677 (14/1613458)	0.994	30	pd	dlt	dc	C25
	c.4421T>C	p.(Leu1474Pro)	44	rs200302125	missense	PubMed: 31477057	BS1, BS2, BP6, PP5, PP3	P(1);LP(4); VUS(13); LB(2);B(1)	N/a	Hypomorphic	0.004396 (7096/1614166)	0.788	24	N/a	pd	pd	N/a
	c.520G>A	p.(Gly174Arg)	19	rs1014839148	Gly subs.▲	PubMed: 37097554	PM1, PM2, PM5, PP3	P(1);LP(3); VUS(3)	LP	LP	0.000008059 (13/1613190)	0.959	32	pd	dlt	dc	C0
	c.2711G>T	p.(Gly904Val)	31	rs1174417447	Gly subs.▲	Novel	PM1, PM2, PM5, PP3	VUS(1)	LP	LB	N/a	0.958	24	pd	dlt	dc	C0
	c.4717G>A	p.(Gly1573Ser)	35	rs761262391	Gly subs.	Novel	PM2, PP3	VUS(1)	LP	LB	0.000008055 (13/1613978)	0.670	25	pd	dlt	plm	C15
	c.416G>A	p.(Gly139Glu)	37	rs2125911862	Gly subs.▲	Novel	PM1, PM2, PM5, PP3	VUS(1)	LP	LP	0.00000062 (1/1612700)	0.979	30	pd	dlt	dc	C15
	c.593G>T	p.(Gly198Val)	39	rs2125924674	Gly subs.▲	Novel	PM1, PM2, PP3	VUS(1)	LP	LP	N/a	0.963	29	pd	dlt	dc	C65
	c.2188G>C	p.(Gly730Arg)	43	rs2106147444	Gly subs.▲	Novel	PM1, PM2, PP3	VUS(1)	LP	LB	N/a	0.963		-	dlt	dc	C0
	c.4702C>T	p.(Pro1568Ser)	23	rs2073644777	missense	Novel	PM2, PP3	VUS(2)	VUS	P	0.000002478 (4/1614174)	0.623	24	pd	dlt	dc	C65
	c.3247G>C	p.(Gly1083Arg)	24	rs2106226509	Gly subs.▲	Novel	PM1, PM2, PM5, PP3.	VUS(1)	VUS	P	N/a	0.985	26	pd	dlt	dc	C0
	c.1021C>T	p.(Arg341Cys)	38	rs778166354	missense▲	Novel	PM1, PM2	VUS(3)	VUS	LP	0.00001363 (22/1613526)	0.467	28	pd	dlt	plm	C15

AlignGVD: C0: least likely to interfere with function, C15: likely benign, C25: uncertain significance, C65: most likely to interfere with function; Gly subs –missense glycine substitution pd-probably damaging; dlt – deleterious; dc – disease causing; plm – polymorphism; bn – benign variant; tlt – tolerated; pat – pathological; N/a – not applicable; chr – chromosome; PP2 – PolyPhen-2; MT – MutationTester; DB – database; ; P –

pathogenic; LP – likely pathogenic; VUS – variant of uncertain significance; LB – likely benign; B – benign; ▲ - substitution in collagenous domain. NOTE: Novel genetic variants were published by us in PubMed ID: 35419377.

Table S2. General characteristics of 48 individuals with ADAS: heterozygous variants in *COL4A4* and *COL4A3*

ID	Family No	Member, gender	Age at diagnosis	Gene	Variant No	Nucleotide change	Protein change	Coding impact
A4106289	62	I-M	46	*COL4A4*	10	c.1579G>T	p.(Gly527Cys)	Missense Gly substitution
A4104793	47	I-M	20					
A4204591	45	I-F	32	*COL4A4*	20	c.-101-4A>G	p.(?)	Splice site
A4252247	22	I-M	18	*COL4A4*	25	c.1389del	p.(Asn464Thrfs*7)	Frameshift
A42555104	55	I-M	41					
A2555105		II-M	12					
A42555106		III-F	8					
A4282553	25	I-F	62	*COL4A4*	28	c.1820C>T	p.(Ala607Val)	Missense non-Gly substitution
A4283570	35	I-F	33					
A4424894	48	I-F	70	*COL4A4*	42	c.3451G>A	p.(Gly1151Arg)	Missense Gly substitution
A453671	63	I-F	66	*COL4A4*	5	c.657+2dup	p.(?)	Unknown
A476197	61	I-F	31	*COL4A4*	7	c.4151C>T	p.(Ala1384Val)	Missense non-Gly substitution
A49717	7	I-F	36	*COL4A4*	9	c.594+1G>A	-	Splice site
A49737		II-F	61					
A49738		III-M	26					
A49775		IV-F	12					
A494387	43	I-M	65					
A4151430	14	I-F	37	*COL4A4*	15	c.5045G>A	p.(Arg1682Gln)	Missense non-Gly substitution
A41558109	58	I-F	58					
A41654103	54	I-M	38	*COL4A4*	16	c.1987G>C	p.(Gly663Arg)	Missense Gly substitution
A41654111		II-M	7					
A4322957	29	I-F	41	*COL4A4*	32	c.2756A>G	p.(Glu919Gly)	Missense non-Gly substitution
A4322976		II-M	3					
A4363463	34	I-M	20	*COL4A4*	36	c.4315G>A	p.(Gly1439Ser)	Missense Gly substitution
A4363469		II-F	47					
A4363477		III-M	10					
A4404082	40	I-M	40	*COL4A4*	40	c.3044G>A	p.(Gly1015Glu)	Missense Gly substitution
A4404085		II-M	2					
A4404086		III-M	3					
A4414284	42	I-F	14	*COL4A4*	41	c.2347G>A	p.(Gly783Arg)	Missense Gly substitution
A44142100		II-M	40					
A44142110		III-M	63					
A3222042	20	I-F	42	*COL4A3*	22	c.898G>A	p.(Gly300Arg)	Missense Gly substitution
A3222043		II-F	17					
A3222044		III-M	8					
A3221872	18	I-M	31					
A3292654	26	I-F	33	*COL4A3*	29	c.3499G>A	p.(Gly1167Arg)	Missense Gly substitution
A32957108	57	I-F	56					
A3445098	50	I-M	34	*COL4A3*	44	c.4421T>C	p.(Leu1474Pro)	Missense non-Gly substitution
A34460114	60	I-M	45					
A3191736	17	I-F	35	*COL4A3*	19	c.520G>A	p.(Gly174Arg)	Missense Gly substitution
A3191774		II-M	7					
A3312856	28	I-M	31	*COL4A3*	31	c.2711G>T	p.(Gly904Val)	Missense Gly substitution
A3353268	32	I-F	1	*COL4A3*	35	c.4717G>A	p.(Gly1573Ser)	Missense Gly substitution
A3353296		II-M	31					
A3373665	36	I-F	63	*COL4A3*	37	c.416G>A	p.(Gly139Glu)	Missense Gly substitution
A339398	39	I-M	54	*COL4A3*	39	c.593G>T	p.(Gly198Val)	Missense Gly substitution
A3434995	49	I-F	42	*COL4A3*	43	c.2188G>C	p.(Gly730Arg)	Missense Gly substitution

Gly – glycine; F- female; M – male; No – number

Table S3. Genotype-phenotype correlation in 48 individuals with ADAS: heterozygous variants in *COL4A4* and *COL4A3*

Gene	Variant	Var No	Fam No., member, gender	Age at diagnosis	Hematuria	Pro, amount, (age at detection, y.o.)	CKD stage	KF- age at KF	Ocular ab.	Hearing ab.
COL4A4	p.Gly527Cys (c.1579G>T)	10	62-I-M	46	+	none	I	none	+	none
			47-I-M	20	+	none	I	none	none	none
	c.-101-4A>G	20	45-I-F	32	+	++ (27)	I	none	none	none
	p.Asn464Thrfs*7 (c.1389del)	25	22-I-M	18	+	+ (19)	I	none	none	none
			55-I-M	41	+	+ (36)	I	none	none	none
			55-II-M	12	+	none	I	none	none	none
			55-III-F	8	+	none	I	none	none	N/d
	p.Ala607Val (c.1820C>T)	28	25-I-F	62	+	+ (61)	IIIa	none	none	none
			35-I-F	33	+	+ (26)	I	none	none	none
	p.Gly1151Arg (c.3451G>A)	42	48-I-F	70	+	+ (62)	IIIb	none	none	none
	c.657+2dup	5	63-I-F	66	+	none	I	none	none	+
	p.Ala1384Val (c.4151C>T)	7	61-I-F	31	+	none	I	none	N/d	N/d
	c.594+1G>A	9	7-II-F	36	+	+ (29)	I	none	+	+
			7-III-F	61	+	++ (53)	V	+ (55)	none	none
			7-IV-M	26	+	none	I	none	none	none
			7-V-F	12	+	+ (12)	I	none	+	none
			43-I-M	65	+	+ (65)	IV	none	none	none
	p.Arg1682Gln (c.5045G>A)[a]	15	14-I-F	37	+	+++ (37)	II	none	N/d	+
			58-I-F	58	+	none	II	none	N/d	N/d
	p.Gly663Arg (c.1987G>C)	16	54-I-M	38	+	none	I	none	none	none
			54-II-F	7	+	none	I	none	+	none
	p.Glu919Gly (c.2756A>G)	32	29-I-F	41	+	+ (30)	I	none	none	none
			29-II-M	3	+	none	I	none	none	none
	p.Gly1439Ser (c.4315G>A)	36	34-I-M	20	+	+ (20)	I	none	none	none
			34-II-F	47	+	none	I	none	none	+
			34-III-M	10	+	none	II	none	none	none
	p.Gly1015Glu (c.3044G>A)	40	40-I-M	40	+	none	I	none	none	none
			40-II-M	2	+	none	I	none	+	none
			40-III-M	3	+	none	I	none	+	none
	p.Gly783Arg (c.2347G>A)	41	42-I-F	14	+	none	I	none	none	none
			42-II-M	40	+	+ (30)	I	none	none	none
			42-III-M	63	+	++ (61)	IIIb	none	N/d	N/d
COL4A3	p.Gly300Arg (c.898G>A)	22	20-I-F	42	+	none	II	none	none	none
			20-II-F	17	+	none	I	none	none	none
			20-III-M	8	+	+ (3)	I	none	none	none
			18-I-M	31	+	none	I	none	none	+
	p.Gly1167Arg (c.3499G>A)	29	26-I-F	33	+	+ (34)	I	none	N/d	none
			57-I-F	57	+	+ (37)	II	none	none	+
	p.Leu1474Pro (c.4421T>C)	44	50-I-M	34	+	+ (33)	I	none	none	none
			60-I-M	45	+	++ (30)	V	+ (44)	none	+
	p.Gly174Arg (c.520G>A)	19	17-I-F	35	+	++ (29)	II	none	none	none
			17-II-M	7	+	none	I	none	none	none
	p.Gly904Val (c.520G>A)	31	28-I-M	31	+	+ (15)	I	none	none	none
	p.Gly1573Ser c.4717G>A	35	32-I-F	1	+	+ (1)	I	none	+	none
			32-II-M	31	+	none	I	none	N/d	N/d
	p.Gly139Glu (c.416G>A)	37	36-I-F	63	+	none	II	none	N/d	none
	p.Gly198Val (c.593G>T)	39	39-I-M	54	+	+ (26)	I	none	none	+
	p.Gly730Arg (c.2188G>C)	43	49-I-F	42	+	++ (42)	I	none	none	none

Var – variant; No – number; Fam – family; M – male; F – female; Pro – proteinuria; y.o. – years of age; CKD – chronic kidney disease; KF – kidney failure; ab. – abnormalities; N/d – no data. Proteinuria: +: PCR 15-49 mg/mmol, ++: PCR 50-299 mg/mmol, +++: ≥300 mg/mmol for adults, ≥200 mg/mmol for children

Table S4. Kidney biopsy findings in individuals with ADAS: heterozygous *COL4A3* and *COL4A4* variants

Variant	Var No	Fam No., member, gender	KB (age at KB)	Interstitial foam cells	FSGS	Unspecific Ig M and C3 deposits in IF	IgA immunoglobulins in IF	Thin GBM	Thickened and lamellated GBM	FPE
***COL4A4* gene**										
p.Gly527Cys (c.1579G>T)	10	62-I-M	none	N/d	N/d	N/d	N/d	N/d	N/d	N/d
		47-I-M	none	N/d	N/d	N/d	N/d	N/d	N/d	N/d
c.-101-4A>G	20	45-I-F	+ (31)	-	-	+	-	+	-	+
p.Asn464Thrfs*7 (c.1389del)	25	22-I-M	+ (17)	-	-	-	-	+	-	+
		55-I-M	+ (36)	-	-	-	-	+	-	+
		55-II-M	none	N/d	N/d	N/d	N/d	N/d	N/d	N/d
		55-III-F	none	N/d	N/d	N/d	N/d	N/d	N/d	N/d
p.Ala607Val (c.1820C>T)	28	25-I-F	+ (61)	-	+	-	-	+	+	+
		35-I-F	+ (31)	-	-	-	-	+	-	+
p.Gly1151Arg (c.3451G>A)	42	48-I-F	+ (69)	-	+	-	+	+	+	+
c.657+2dup	5	63-I-F	+ (65)	-	-	-	-	+	-	+
p.Ala1384Val (c.4151C>T)	7	61-I-F	none	N/d	N/d	N/d	N/d	N/d	N/d	N/d
c.594+1G>A	9	7-II-F	none	N/d	N/d	N/d	N/d	N/d	N/d	N/d
		7-III-F	+ (53)	N/d	+	N/d	N/d	N/d	N/d	N/d
		7-IV-M	none	N/d	N/d	N/d	N/d	N/d	N/d	N/d
		7-V-F	none	N/d	N/d	N/d	N/d	N/d	N/d	N/d
		43-I-M	+ (64)	-	+	-	-	+	+	+
p.Arg1682Gln (c.5045G>A)	15	14-I-F	+ (36)	-	+	+	-	+	+	+
		58-I-F	+ (57)	-	-	-	-	+	-	+
p.Gly663Arg (c.1987G>C)	16	54-I-M	none	N/d	N/d	N/d	N/d	N/d	N/d	N/d
		54-II-F	none	N/d	N/d	N/d	N/d	N/d	N/d	N/d
p.Glu919Gly (c.2756A>G)	32	29-I-F	+ (40)	-	-	-	-	+	-	+
		29-II-M	none	N/d	N/d	N/d	N/d	N/d	N/d	N/d
p.Gly1439Ser (c.4315G>A)	36	34-I-M	none	N/d	N/d	N/d	N/d	N/d	N/d	N/d
		34-II-F	none	N/d	N/d	N/d	N/d	N/d	N/d	N/d
		34-III-M	none	N/d	N/d	N/d	N/d	N/d	N/d	N/d
p.Gly1015Glu (c.3044G>A)	40	40-I-M	none	N/d	N/d	N/d	N/d	N/d	N/d	N/d
		40-II-M	none	N/d	N/d	N/d	N/d	N/d	N/d	N/d
		40-III-M	none	N/d	N/d	N/d	N/d	N/d	N/d	N/d
p.Gly783Arg (c.2347G>A)	41	42-I-F	none	N/d	N/d	N/d	N/d	N/d	N/d	N/d
		42-II-M	none	N/d	N/d	N/d	N/d	N/d	N/d	N/d
		42-III-M	none	N/d	N/d	N/d	N/d	N/d	N/d	N/d
***COL4A3* gene**										
p.Gly300Arg (c.898G>A)	22	20-I-F	none	N/d	N/d	N/d	N/d	N/d	N/d	N/d
		20-II-F	none	N/d	N/d	N/d	N/d	N/d	N/d	N/d
		20-III-M	none	N/d	N/d	N/d	N/d	N/d	N/d	N/d
		18-I-M	+ (30)	-	-	-	-	+	-	+
p.Gly1167Arg (c.3499G>A)	29	26-I-F	+ (32)	-	-	-	-	+	-	+
		57-I-F	none	N/d	N/d	N/d	N/d	N/d	N/d	N/d
p.Leu1474Pro (c.4421T>C)	44	50-I-M	+ (33)	-	-	-	+	+	+	+
		60-I-M	+ (30)	N/d	+	-	-	N/d	N/d	N/d
p.Gly174Arg (c.520G>A)	19	17-I-F	+ (31)	-	-	-	-	+	+	+
		17-II-M	none	N/d	N/d	N/d	N/d	N/d	N/d	N/d
p.Gly904Val (c.520G>A)	31	28-I-M	+ (30)	-	-	-	-	+	-	+
p.Gly1573Ser c.4717G>A	35	32-I-F	none	N/d	N/d	N/d	N/d	N/d	N/d	N/d
		32-II-M	none	N/d	N/d	N/d	N/d	N/d	N/d	N/d
p.Gly139Glu (c.416G>A)	37	36-I-F	+ (61)	-	+	+	+	+	-	+
p.Gly198Val (c.593G>T)	39	39-I-M	+ (53)	-	+	-	-	+	-	+
p.Gly730Arg (c.2188G>C)	43	49-I-F	+ (42)	-	-	-	-	+	-	+

Var – variant; No – number; Fam – family; M – male; F – female; N/d – no data; FSGS – focal segmental glomerulosclerosis; IgM – immunoglobulin M; IgA – immunoglobulin A; KB – kidney biopsy; FPE – foot process effacement

Table S5. General characteristics of 4 individuals with ARAS: compound heterozygous and homozygous variants

ID	Family no	Member, gender	Age at diagnosis	Gene	Variant No	Nucleotide change	Protein change	Type of ARAS	Reported or novel variant
A4/49716	7; 62	I-M	7	*COL4A4*	9	c.594+1G>A	-	Compound het	Novel
				COL4A4	10	c.1579G>T	p.Gly527Cys		Reported
A3/3232145	21	I-F	34	*COL4A3*	23	c.4702C>T	p.Pro1568Ser	Compound het	Novel
				COL4A3	24	c.3247G>C	p.Gly1083Arg		Novel
A3/3232146		II-F	34	*COL4A3*	23	c.4702C>T	p.Pro1568Ser	Compound het	Novel
				COL4A3	24	c.3247G>C	p.Gly1083Arg		Novel
A4/4/283867	38	I-F	32	*COL4A4*	28	c.1820C>T	p.Ala607Val	Homo	Reported

No – number; ARAS – autosomal recessive Alport syndrome; het – heterozygous; homo – homozygous; F – female; M – male

Table S6. Genotype-phenotype correlation in 4 individuals with ARAS: compound heterozygous and homozygous variants

Gene	Variant	Var No	Fam No., member, gender, ID	Age at diagnosis	Hematuria	Proteinuria (age at detection, amount)	CKD stage	KF- age at kidney failure	Ocular ab.	Hearing ab.
COL4A4	c.594+1G>A	9	7-62-I-M (A4/49716)[y]	7	+	++ (6)	I	none	+	+
	p.Gly527Cys (c.1579G>T)	10								
	p.Ala607Val (c.1820C>T) x2	28	38-I-F A4/4/283867[xy]	32	+	+++ (22)	V	+ (32)	+	+
COL4A3	p.Pro1568Ser (c.4702C>T)	23	21-I-F A3/3232145	34	+	+++ (23)	V	+ (33)	none	+
			21-I-F A3/3232146	34	+	+++ (30)	IIIb	none	none	+
	p.Gly1083Arg (c.3247G>C)	24	21-I-F A3/3232145	34	Sharing phenotype with p.Pro1568Ser (c.4702C>T) variant					
			21-I-F A3/3232146	34	Sharing phenotype with p.Pro1568Ser (c.4702C>T) variant					

Var – variant, No – number, Fam – family, M – male, F – female, Pro – proteinuria, y.o. – years of age, CKD – chronic kidney disease, KF – kidney failure, ab. – abnormalities. Proteinuria: +: PCR 15-49 mg/mmol, ++: PCR 50-299 mg/mmol, +++: ≥300 mg/mmol for adults, ≥200 mg/mmol for children

Table S7. Kidney biopsy findings in individuals with ARAS: compound heterozygous and homozygous variants

Gene	Variant	Var No	Fam No., member, gender, ID	KB (age at KB)	Foam cells	FSGS	Unspecific Ig M and C3 deposits in IF	IgA in IF	Thin GBM	Thickened and lamellated GBM	FPE
COL4A4	c.594+1G>A	9	7-62-I-M (A4/49716)	none	N/d	N/d	N/d	N/d	N/d	N/d	N/d
	p.Gly527Cys (c.1579G>T)	10									
	p.Ala607Val (c.1820C>T) x2	28	38-I-F A4/4/283867	+ (26)	-	-	-	-	+	+	+
COL4A3	p.Pro1568Ser (c.4702C>T)	23	21-I-F A3/3232145	+ (31)	-	+	+	-	+	+	+
			21-II-F A3/3232146	+ (32)	+	+	-	-	+	+	+
	p.Gly1083Arg (c.3247G>C)	24	21-I-F A3/3232145	Sharing phenotype with p.Pro1568Ser (c.4702C>T) variant							
			21-II-F A3/3232146	Sharing phenotype with p.Pro1568Ser (c.4702C>T) variant							

Var – variant; No – number; Fam – family; M – male; F – female; N/d – no data; FSGS – focal segmental glomerulosclerosis; IgM – immunoglobulin M; IgA – immunoglobulin A; KB- kidney biopsy; FPE – foot process effacement

Table S8. General characteristics of 4 individuals with digenic AS

ID	Famil	Member,	Age at	Gene	Varia	Nucleotide change	Protein change	Type of Digenic AS	Reported or novel variant

	y no	gender	diagnosis		nt No				
A5/46512	5	I-F	14	COL4A5	6	c.1417_1418del	p.Val473Glufs*3 Frameshift	X-linked similar inheritance	Novel
				COL4A4	7	c.4151C>T	p.Ala1384Val		Novel
A5/412818	8	I-F	39	COL4A4	11	c.2996G>A	p.Gly999Glu	X-linked similar inheritance	Reported
				COL4A5	12	c.1871G>A	p.Gly624Asp		Reported
A5/4262450	24	I-F	26	COL4A5	26	c.1374delinsTT	p.Pro459Serfs*6	X-linked similar inheritance	Novel
				COL4A4	27	c.4910G>A	p.Arg1637Gln		Novel
A3/438376	37	I-M	42	COL4A3	38	c.1021C>T	p.Arg341Cys	ADAS similar inheritance	Novel
				COL4A4	20	c.-101-4A>G	-		Reported

No – number; ADAS – autosomal dominant Alport syndrome; F – female; M – male.

Table S9. Genotype-phenotype correlation in 4 individuals with digenic AS

Gene	Variant	Var. No	Fam No., member, gender, ID	Age at diagnosis	Hematuria	Pro (age at detection, amount)	CKD stage	KF- age at kidney failure	Ocular ab.	Hearing ab.
COL4A5	p.Val473Glufs*3 (c.1417_1418del)	6	5-I-F A5/46512[y]	14	+	+ (14)	I	none	none	none
COL4A4	p.Ala1384Val (c.4151C>T)	7								
COL4A4	p.Gly999Glu (c.2996G>A)	11	8-I-F A5/412818[xy]	39	+	++ (22)	II	none	+	+
COL4A5	p.Gly624Asp (c.1871G>A)	12								
COL4A5	p.Pro459Serfs*6 (c.1374delinsTT)	26	24-I-F A5/4262450[y]	26	+	+ (16)	I	none	none	none
COL4A4	p.Arg1637Gln (c.4910G>A)	27								
COL4A3	p.Arg341Cys (c.1021C>T)	38	37-I-M A3/438376	42	+	+ (35)	I	none	none	none
COL4A4	c.-101-4A>G	20								

Var – variant; No – number; Fam – family; M – male; F – female; Pro – proteinuria; y.o. – years of age; CKD – chronic kidney disease; KF – kidney failure; ab. – abnormalities. Proteinuria: +: PCR 15-49 mg/mmol, ++: PCR 50-299 mg/mmol, +++: ≥300 mg/mmol for adults, ≥200 mg/mmol for children

Table S10. Kidney biopsy findings in 3 of 4 individuals with digenic AS

Gene	Variant	Variant No	Fam No., member, gender, ID	KB (age at KB)	Foam cells	FSGS	Unspecific IgM and C3 deposits in IF	IgA in IF	Thin GBM	Thickened and lamellated GBM	FPE
COL4A5	p.Val473Glufs*3 (c.1417_1418del)	6	5-I-F A5/46512	+ (14)	-	+	-	-	+	+	+
COL4A4	p.Ala1384Val (c.4151C>T)	7									
COL4A4	p.Gly999Glu (c.2996G>A)	11	8-I-F A5/412818	+ (37)	+	-	-	+	+	+	+
COL4A5	p.Gly624Asp (c.1871G>A)	12									
COL4A5	p.Pro459Serfs*6 (c.1374delinsTT)	26	24-I-F A5/4262450	none	N/d	N/d	N/d	N/d	N/d	N/d	N/d
COL4A4	p.Arg1637Gln (c.4910G>A)	27									
COL4A3	p.Arg341Cys (c.1021C>T)	38	37-I-M A3/438376	+ (40)	-	-	-	-	+	-	+
COL4A4	c.-101-4A>G	20									

Var – variant; No – number; Fam – family; M – male; F – female; N/d – no data; FSGS – focal segmental glomerulosclerosis; IgM – immunoglobulin M; IgA – immunoglobulin A; KB- kidney biopsy; FPE – foot process effacement
